# Evaluating the Effect of Dexmedetomidine on Hemodynamic Status of Patients with Septic Shock Admitted to Intensive Care Unit: A Single-Blind Randomized Controlled Trial

**DOI:** 10.22037/ijpr.2019.112343.13699

**Published:** 2020

**Authors:** Shayesteh Gheibi, Shahram Ala, Fatemeh Heydari, Ebrahim Salehifar, Hamideh Abbaspour kasgari, Siavash Moradi

**Affiliations:** a *Department of Pharmacotherapy, Faculty of Pharmacy, Mazandaran University of Medical Sciences, Sari, Iran. *; b *Department of Anesthesiology and Critical Care Medicine, Imam Khomeini Hospital, Mazandaran University of Medical Sciences, Sari, Iran.*; c *Pharmaceutical Science Research Center, Hemoglobinopathy institute, Sari, Iran. *; d *Department of Clinical pharmacy, Antimicrobial Resistance Research Center, Ghaemshahr Razi hospital, Mazandaran University of Medical Science,sari,Iran. *; e *Directr of Medical Education Development Center, School of Medicine, Mazandaran University of Medical Sciences, Sari, Iran.*

**Keywords:** DXM, Septic Shock, MAP, SBP, DBP, HR

## Abstract

Septic shock, known as the most severe complication of sepsis, is a serious medical condition that can lead to death. Clinical symptoms of sepsis include changes in body temperature in the form of hypothermia or hyperthermia, tachypnea or hyperventilation, tachycardia, leukocytosis or leukopenia, and variations in blood pressure, as well as altered state of consciousness. One of the main problems in septic shock is poor response along with reduced vascular reactivity to vasopressors used to increase blood pressure. Therefore, low vascular response associated with reduced sensitivity or lower number of alpha-1 agonist receptors can result in shock and death. In addition to being the state-of-the-art treatment including volume load and vasopressor, use of alpha-2 agonists *e.g*. dexmedetomidine (DXM) in septic shock can reduce vasopressors needed to restore adequate blood pressure. They can further moderate massive release of endogenous catecholamine. Therefore, the purpose of this study was to investigate the effect of DXM on outcomes of patients with septic shock, especially their needs for vasopressors and impacts on their hemodynamic status. This single-blind randomized controlled trial was performed on a total number of 66 patients with septic shock admitted to the intensive care unit (ICU) of Imam Khomeini Teaching Hospital in the city of Sari, in northern Iran. To this end, DXM (0.6 µg/kg/h) and normal saline (6 mL/kg/h) were infused for 12 h in the study and control groups, respectively. The results revealed that DXM could increase mean arterial pressure (MAP) (*P *= 0.021), systolic blood pressure (SBP) (*P* = 0.002), and reduced heart rate (*P *< 0.001) but diastolic blood pressure (DBP) (*P* =0.32) and norepinephrine dose requirement didn’t change statistically in septic shock patients (*P* = 0.12).

## Introduction

Obtaining enough information about sepsis has been considered as one of the medical science concerns (1). After each inflammation, circulatory disorders including decreased intravascular volume, peripheral vasodilatation, myocardial dysfunction, as well as increased metabolism may also lead to an imbalance between systemic oxygen delivery and oxygen demand, which ultimately induce hypoxia (2). 

Since 2016, sepsis has been termed as a life-threatening organ dysfunction; as Sequential Organ Failure Assessment (SOFA) score > 2, with a mortality of over 10% in hospitals. Clinically, patients with septic shock are described as those requiring a vasopressor to maintain their mean arterial pressure (MAP) of 65 mmHg or more and serum lactate levels greater than 2 mmol/L (> 8 mg/dL) in the absence of a hypovolemia. Individuals with suspected sepsis infection can correspondingly have very poor recovery outcomes if they have at least two of criteria of Quick SOFA (qSOFA) including tachypnea (defined as more or equal breathing 22 per min, hypotension (defined as systolic blood pressure (SBP ≤ 100 mmHg), and altered state of consciousness (3).

In the first phase, systemic inflammatory response can be considered as a result of host responses to bacterial products such as endotoxin. Then, cytokines are activated and cause further physiological disorders. The onset of septic shock is thus characterized by high concentrations of catecholamines in the circulation. Disturbance in sympathetic regulation of the cardiovascular system also indicates that autonomic system impairment produces circulatory failure (4). 

The most common causes of severe sepsis are pneumonia (responsible for more than half of cases) as well as intra-abdominal and urinary tract infections. Even with intensive care, mortality rates in hospitalized septic shock patients have been reported by 80% over the past 30 years. 

Following the administration of norepinephrine, MAP can increase from 65 mmHg to 85 mm Hg; however, systemic oxygen metabolism, skin microcirculatory blood flow, and exudation and visceral perfusion do not significantly improve. Increasing doses of dopamine and dobutamine alone and in combination can be also effective in arterial hypotension treatment. Norepinephrine alone can further increase MAP and systemic vascular resistance and epinephrine can instigate a slight rise in heart rate (5, 6 and 7).

One of the frequently reported problems in septic shock is poor response and reduced vascular reactivity to vasopressors used to increase blood pressure. In this regard, efforts to restore vascular responses with nitric oxide inhibitors or low-dose steroids have been also unsuccessful. Low vascular responses associated with reduced sensitivity and number of alpha-1 agonist receptors can correspondingly result in resistance shock and death. 

Dexmedetomidine (DXM) (*i.e*. a central alpha-2 agonist), as the active isomer of medetomidin, was licensed by the Food and Drug Administration (FDA) in 1999 to sedate patients admitted to intensive care units (ICUs). Accordingly, addition of alpha-2 agonist agents such as clonidine or DXM to common septic shock treatments could reduce vasopressor dose requirements. In other words, alpha-2 agonists can activate receptors in medullary vasomotor center. The second effect also plays an important role in sedation and anti-anxiety of this group, since decreasing the output of noradrenergic neurons from the locus cereulus increases the activity of inhibitory neurons such as gamma aminobutyric acid. Furthermore, several studies have shown that DXM can suppress inflammatory reactions and protect organs in both animals and humans. In major surgical procedures, DXM can also improve blood circulation and reduce mortality.

Clonidine and DXM can also increase venous return accompanied by increased arterial heart rate in sepsis and septic shock leading to maintained arterial blood pressure (6-10). 

With regard to the non-effectiveness of use of common vasopressors in maintaining patients’ hemodynamics, the purpose of this study was to evaluate the effect of DXM on hemodynamic status of patients with septic shock.

## Experimental

This study was a single-blind randomized controlled trial conducted between August 2018 and June 2019 at the ICU of Imam Khomeini Teaching Hospital affiliated to Mazandaran University of Medical Sciences (MAZUMS), Sari, Iran. The study was approved by the Mazandaran University of Ethics Committee (No.IR.MAZUMS.REC.1397.3095), registered in www.IRCT.ir under IRCT No. IRCT20100107003014N22, and financially supported by the Vice-Chancellor’s Office for Research and Technology at MAZUMS with grant No. 3095. Informed consent was also obtained from patients’ legal and personal representatives included in the study.

*Patient selection*

*sample size*

Considering the assumptions, the minimum sample size in each arm was 30 patients, assuming a maximum of 10% fall during the study to 33 patients in each arm.

SD = 2.85 POWER = 80%, CI = 95%, CL = 2 Days,

N = )2K × SD(^2^/d^2^

N = (2 × 7.6 × 2.85) 2/2^2^

N = 33 in each arm

*Participants and setting*

The patients were recruited from Imam Khomeini Teaching Hospital affiliated to MAZUMS. The informed consent was also obtained from all the participants before their recruitment. It should be noted that participants eligible for the study were those meeting diagnostic criteria of septic shock/sepsis (Have at least two qSOFA criteria at the same time, which include a respiratory rate greater than or equal to 22 (bpm), alteration of consciousness, systolic blood pressure less than or equal to 100 mmHg). Moreover, the inclusion criteria were the patients aged over 18 years with MAP above 65 mmHg. In addition, the patients with uncontrolled diabetes mellitus (HbA_1_C > 7%), heart block grade 2 or 3, sick sinus syndrome, history of chronic obstructive pulmonary disease (COPD) (arterial oxygen pressure below 60 mmHg), history of use of beta-blockers, hypovolemia (CVP < 8 cmHg), and sever hepatic failure (markedly elevated transaminase, INR ≥ 1.5, sign of hepatic encephalopathy) were excluded.

*Randomization and intervention*

The patients were recruited in intervention and control arms by simple randomization procedure using a table of random numbers. The patients with hypotension and tissue hypoperfusion in the control group received a crystalloid fluid with the amount of 30 mL/kg, and then in the case of persistent hypotension, norepinephrine (0.01-4 μg/kg/min) was started.

In addition, the second group received DXM at a dose of 0.6 μg/kg/h (Precedex, made by Hospira, USA) for 12 h. DXM vials contained 100 μg of drug which needed to be diluted to reach a concentration of 4 μg/mL. Normal saline (6 mL/kg/h) was also infused for 12 h instead of DXM. Infusion was discontinued in both groups, if heart rate decreased or less equal 60 beat/min if MAP dropped and also this condition did not improve via vasopressor therapy. We followed up patients in day 2 and 7, respectively. In this study, the final analyzer was blinded to the study.

*Primary outcome*

The effect of DXM on hemodynamic status of the patients with septic shock was evaluated for 66 patients. These parameters include SBP, DBP, MAP, HR, and dose of NE requirement.

*Response assessment*

Changes in heart rate, SBP and diastolic blood pressure (DBP), MAP, as well as norepinephrine dose requirement during 12 h of the study were evaluated.

*Statistical analysis*

At first, the distribution of the data was plotted by a histogram and explained by Kolmogorov-Smirnov test. Then, quantitative data were described while calculating mean standard deviation (SD) or median (quartile range) and qualitative data were controlled through percentage frequency. According to the distribution of the data, the means or medians of the quantitative variables were compared using independent *t-*test or its nonparametric equivalent, *i.e*. Mann-Whiney* U*-test. Chi-square test was similarly utilized to compare the frequency of qualitative variables. For the repeated measured value we used “Repeated measure ANOVA”. It should be noted that analysis in this study was of intention-to-treat type and two-tailed *P*-value less than 0.05 was considered statistically significant in all cases. Descriptive and statistical analyses were performed using IBM SPSS Statistics software (version 21).

## Results

**Figure 1:**
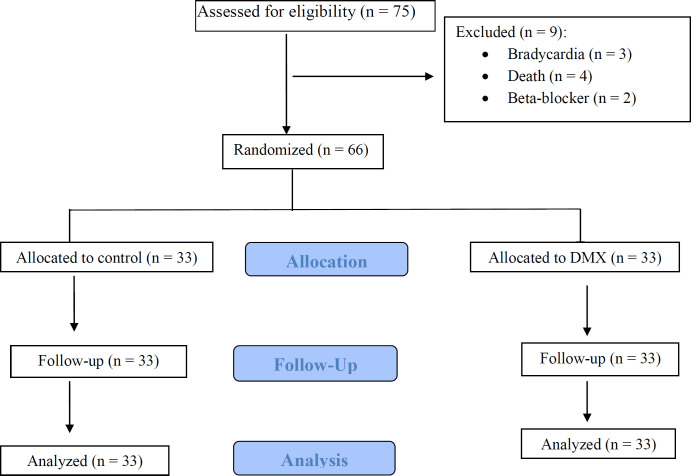
Diagram of participants (according to CONSORT 2010 guidelines)

Study Participants of 75 patients who were screened for eligibility, showed that 9 patients were excluded and 66 were enrolled. From 66 patients (33 in each study arm) who were randomized in the two groups nobody was lost during allocation and follow up in the case and control groups. Finally, the data analysis was performed on 33 patients, who completed the study (Figure 1). Baseline demographic and clinical characteristics of DXM and control groups approximately were similar (Table 1). Also, laboratory values and physiological variables did not differ significantly between the two groups at baseline and during the study period.

*Hemodynamic evaluation of patients*

**Figure 2:**
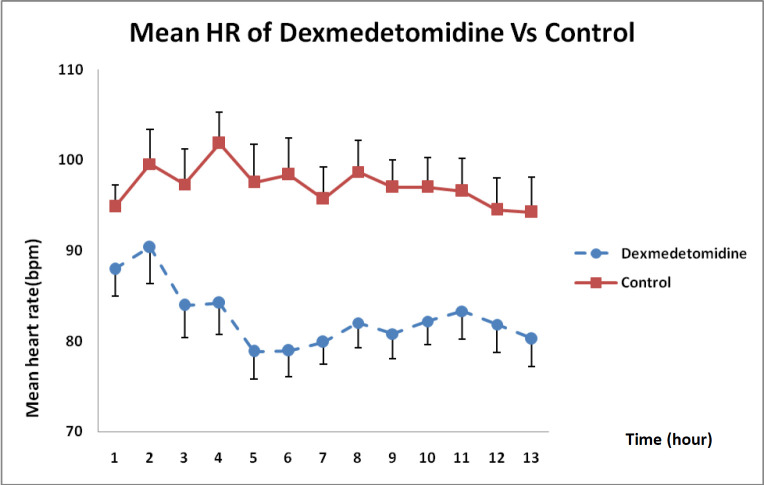
Heart rate changes in* DXM *and control groups over 12 h (mean ± SE) (*P* < 0.01)

As shown in Figure 2, DXM caused a higher reduction in heart rate than that in the control group receiving normal saline during the 12 h infusion and this difference was statistically significant (*P* < 0.01).

**Figure 3:**
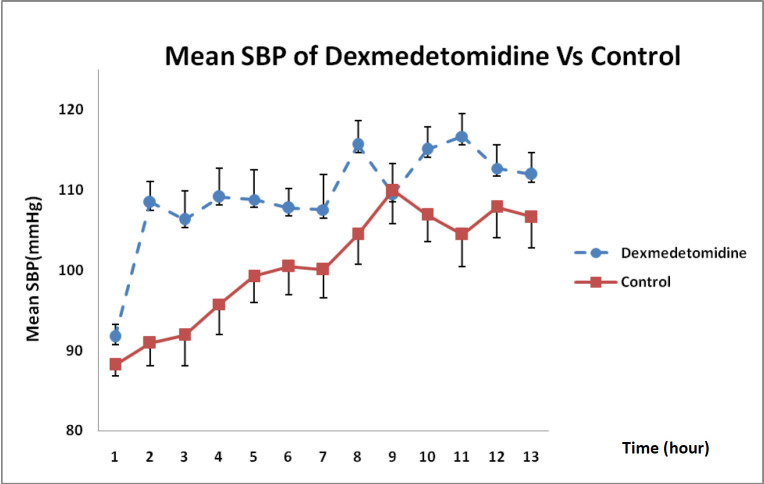
SBP changes in DXM and control groups over 12 h (mean ± SE) (*P *= 0.002)

Considering the state of SBP changes over 12 h, illustrated in Figure 3, DXM could increase SBP more than that in the control group and this difference was statistically significant in each hour except at 9 h (*P* = 0.002). 

**Figure 4:**
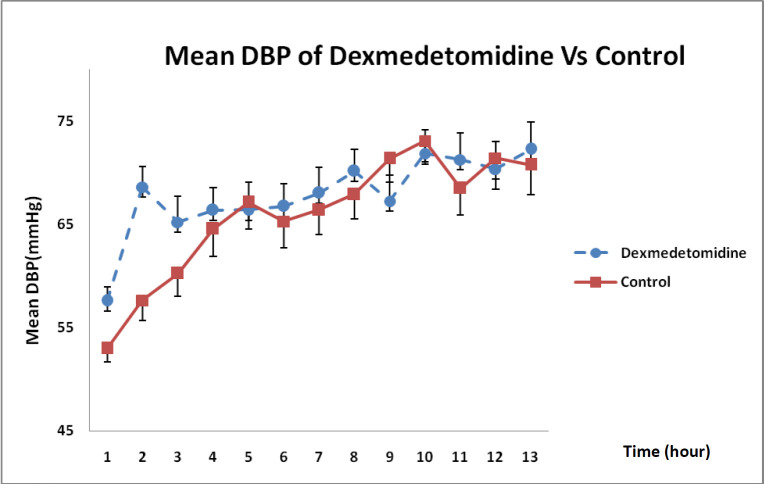
DBP changes in DXM and control groups over 12 h (mean ± SE) (*P* = 0.32)

With regard to the state of DBP changes during 12-h infusion in Figure 4, it was revealed that DXM could enhance DBP more than that in the control group even though such an increase was not statistically significant (*P* = 0.32).

**Figure 5:**
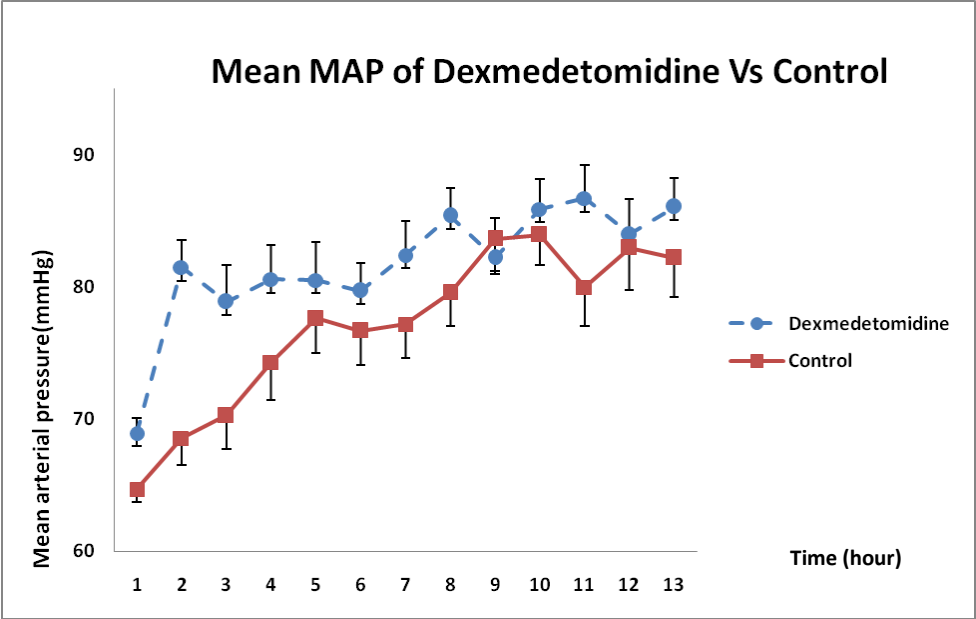
MAP changes in DXM and control groups over 12 h (mean ± SE) (*P *= 0.021)

In Figure 5, MAP changes between both groups indicated that DXM infusion during 12 h had significantly boosted MAP compared with that in the control group and such a difference was statistically significant (*P* = 0.021).

**Figure 6:**
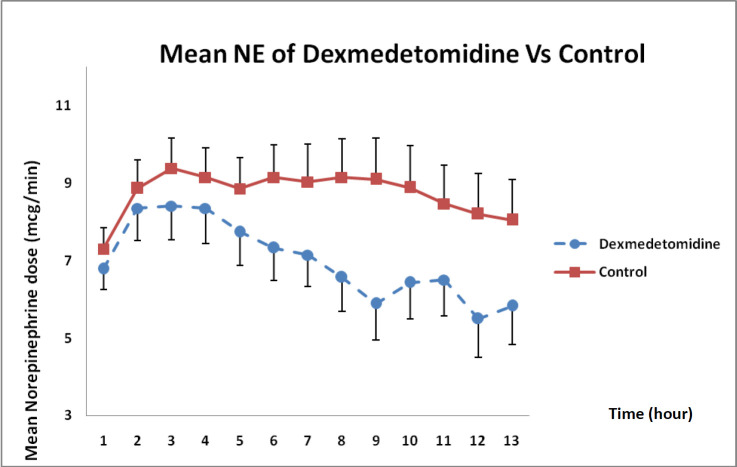
Mean norepinephrine dose changes in DXM and control groups over 12 h (mean ± SE) (*P* = 0.12)

As presented in Figure 6, norepinephrine dose requirements between both groups showed that the dose had decreased during 12 h compared with that in the control group but such a difference was not statistically significant (*P* = 0.12).

The patients were followed up on days 2 and 7 after septic shock using SOFA criteria and no significant differences were observed between the two groups (Table 2).

## Discussion

Although our patients received morphine and midazolam as sedatives and analgesics before entering the study, they were discontinued due to the FDA-approved analgesic effects of DXM and only DXM continued. DMX is known as a lipophilic drug, with high distribution volume and a half-life about 6 min. Compared with clonidine, DXM is an alpha-2 agonist with an affinity of more than 8-fold to adrenergic receptors. It also has sedative, analgesic, and anti-anxiety effects. In this respect, phase 3 clinical trials had demonstrated that DXM with a dose of 0.2-0.7 µg/kg/h could have sedative and analgesic effects on the patients undergoing post-surgical ventilation, without respiratory depression after separation from the machine. The side effects mostly observed for this drug were also nausea, hypotension, and bradycardia. In the studies investigating pharmacokinetics of DXM in the patients with severe renal insufficiency (*i.e*. creatinine clearance of less than 30), the half-life of the elimination had decreased. The mean half-life of the drug in healthy people had been by 2.5 h; however, in the patients with hepatic failure, it had been prolonged. As a result, the dose of this drug in the patients with septic shock needed to be reduced in case of liver involvement, depending on the degree of hepatic failure (7, 10 and 11).

In a research carried out by Marcos *et al*. (2015), on Syrian golden hamsters, the groups had been evaluated in terms of microcirculatory parameters, venous leukocyte-endothelial reactions, as well as correlation between intravenous leukocyte-endothelial interactions and capillary perfusion changes, variations in MAP, and heart rate; suggesting that DXM had reduced adhesion and circulation of leukocytes and capillary density. 

On the other hand, DXM had lowered heart rate without any significant drop in MAP (12). In the present study, DXM increased MAP due to its agonistic effects on alpha-2 receptors although it decreased heart rate.

In another study by Yu *et al*. (2014) examining the difference between effects of propofol and DXM with 10 mg/mL concentrations on the preload of 16 endotoxemic rabbits receiving norepinephrine, it had been confirmed that propofol had increased heart rate, without affecting contractility of myocardium and vascular resistance; in contrast, DXM had augmented cardiac contractility and vascular resistance at high doses (13). As we detected in our study, the DXM increased MAP by affecting on systemic vascular resistance.

In the investigation by Geloen, use of clonidine and DXM in septic shock patients had correspondingly improved venous return along with heart rate, leading to maintained MAP. In the patients undergoing liver transplantation treated with the given drugs, an increase in DBP had been also associated with a decrease in heart rate and a reduced need for vasopressors (6).

However, the study by Penttilä had demonstrated that DXM had lowered SBP and DBP in healthy males and the etiology had remained still unclear because of the alteration of sensitivity of the receptors in septic shock. Due to the effects of DXM on presynaptic alpha-1 receptors, SBP and DBP had increased in the present study.

Besides, in a case study conducted by Leroy *et al.,* (2017) on a 29-week-old child with septic shock secondary to necrotizing enterocolitis, clonidine had been initiated at a dose of 1 μg/kg/h continued by infusion for 13 h after taking the initial steps and the results had revealed that clonidine could improve blood circulation and subsequently decrease norepinephrine dose requirement (14, 15). In the present study, DXM by 0.6 µg/kg/h concentration during 12-h infusion could reduce norepinephrine dose requirement but the rate of reduction of norepinephrine was not sufficient to completely eliminate it and there was still a trace of dependence on vasopressors.

In 1993, Dyck *et al*., had performed a study on pharmacokinetics of DXM for intravenous and intramuscular administration, with a dose of 2 μg/kg in healthy volunteers, and showed that the percentage of metabolic biomarker of DXM in comparison with the intravenous administered dose, had been by 73 ± 11%. After intramascular injection, average arrival time to maximum concentration had been 12 min (at a range of 2-60 min) and average maximum concentration had been 81.2 ± 0.27 ng/mL. After intravenous administration of DXM, dual changes in blood pressure had been observed. During the first 5 min of intravenous injection by 2 μg/kg, MAP had increased by 22% and heart rate had decreased by 27% from baseline. Within 4 h after injection, MAP had also decreased by 20% from baseline and heart rate had reached less than 5% of the initial values. The hemodynamic profile had not shown any acute changes after intramuscular administration. Within 4 h after intramuscular injection, MAP and heart rate had subsequently decreased by 20% and 10%, respectively (16). In the present study, intravenous infusion of DXM also reduced heart rate at 3, 5, 6, and 9 h and increased MAP at 2, 8 and 11 h. It should be noted that the effect of the lowest dose was mediated mainly by arteriolar vasoconstriction, probably due to its cumulative effect.

Moreover, in a study by Lin *et al*., (2009), the effects of simultaneous administration of morphine and DXM for anxiolytic effects had been reported and the results had suggested that morphine at a dose of 1 mg/mL accompanied by 5 µg/mL of DXM had reduced the dose of morphine by 29% within 24 h despite an increase in the analgesic effect from the second hour after surgery.

MAP had decreased and pulse rate was higher in the group receiving DXM. Within 4 to 24 h, the incidence of nausea had been lower in DXM group. In general, in this study, performed on 100 women undergoing hysterectomy, the use of this drug had not induced bradycardia or hypotension, respiratory depression, and excessive sedation in the patients (17). 

In this study, since individuals with septic shock had failed to have spontaneous oral nutrition, they had been mainly fed through nasogastric tube and nausea had been assessed by the amount of fluid returned through it. Accordingly, the results revealed that the drug had not increased nausea in these patients compared with the control group. 

Although DXM couldn’t have significant changes in APACHE II, SOFA, hospital and ICU stay, this drug could increase the MAP that is very important in septic shock patients.

**Table 1:** Demographic and clinical characteristics of patients at baseline

Data are reported as mean ± SD or as number (percentages).* P*-values were obtained by *chi-square*-*test or Fisher’s exact test as appropriate*, *† Independent sampled* t-*test*, ‡ *Mann-Whitney* U*-test with significance set at* P *< 0.05.

*GCS, *Glasgow Coma Scale*; *APACHE II*, *Acute Physiologic and Chronic Health Evaluation; Respiratory: including ventilator-associated pneumonia (VAP)*, *acute respiratory distress syndrome* (*ARDS)*, *and bronchiectasis; CNS such as intracerebral hemorrhage (ICH), subarachnoid hemorrhage (SAH)*,* and brain aneurysm ; cancers including brain tumor and breast cancer types; internal including pyelonephritis, mediastinitis, urosepsis, and peritonitis, Other:major vascular surgery or orthopedic surgeries

**Table 2:** Changes of SOFA score at baseline, 2^nd ^and 7^th ^of ICU admission in DXM* vs. *control group

**SOFA score**	**Mean ± SD control**	**Mean ±** **.** **SD DXM**	***p*** **-value**
Baseline	9.3 ±2.8	9.5 ±.2.4	0.71
Second day	8.5 ± 3.1	8.1 ± 3	0.9
Seventh day	4.2 ±4.5	5 ± 4.1	0.3

## Conclusion

The present study showed that DXM at a 0.6 µg/kg/h dose during a 12-h infusion had increased SBP, DBP, and MAP, and consequently decreased heart rate and norepinepine dose requirements. Given that there might be numerous mechanisms involved in septic shock, it was suggested to administer DXM to a larger number of septic shock patients with different doses and infusion durations to obtain more definitive conclusions.
